# Modeling synergistic effects by using general Hill-type response surfaces describing drug interactions

**DOI:** 10.1038/s41598-022-13469-7

**Published:** 2022-06-22

**Authors:** Michael Schindler

**Affiliations:** Institute of Theoretical Chemistry, Ruhr-University Bochum, 44780 Bochum, Germany

**Keywords:** Computational biology and bioinformatics, Molecular medicine, Mathematics and computing

## Abstract

The classification of effects caused by mixtures of agents as synergistic, antagonistic or additive depends critically on the reference model of ’null interaction’. Two main approaches to describe co-operative effects are currently in use, the Additive Dose (ADM) or concentration addition (CA) and the Multiplicative Survival (MSM) or independent action (IA) models. Recently we proposed an approach which describes ’zero-interaction’ surfaces based on the only requirement that simultaneous administration of different drugs leads to Hill-type response surfaces, which are solutions of the underlying logistic differential equations. No further assumptions, neither on mechanisms of action nor on limitations of parameter combinations are required. This defines—and limits—the application range of our approach. Resting on the same principle, we extend this ansatz in the present paper in order to describe deviations from the reference surface by generalized Hill-type functions. To this end we introduce two types of parameters, perturbations of the pure drug Hill-parameters and interaction parameters that account for n-tuple interactions between all components of a mixture. The resulting ‘full-interaction’ response surface is a valid solution of the basic partial differential equation (PDE), satisfying appropriate boundary conditions. This is true irrespective of its actual functional form, as within our framework the number of parameters is not fixed. We start by fitting the experimental data to the ‘full-interaction’ model with the maximum possible number of parameters. Guided by the fit-statistics, we then gradually remove insignificant parameters until the optimum response surface model is obtained. The ’full-interaction’ Hill response surface ansatz can be applied to mixtures of n compounds with arbitrary Hill parameters including those describing baseline effects. Synergy surfaces, i.e., differences between full- and null-interaction models, are used to identify dose-combinations showing peak synergies. We apply our approach to binary and ternary examples from the literature, which range from mixtures behaving according to the null-interaction model to those showing strong synergistic or antagonistic effects. By comparing ’null-’ and ’full-response’ surfaces we identify those dose-combinations that lead to maximum synergistic or antagonistic effects. In one example we identify both synergistic and antagonistic effects simlutaneously, depending on the dose-ratio of the components. In addition we show that often the number of parameters necessary to describe the response can be reduced without significantly affecting the accuracy. This facilitates an analysis of the synergistic effects by focussing on the main factors causing the deviations from ’null-interaction’.

## Introduction

The observation of synergistic or antagonistic effects after simultaneous administration of mixtures of active ingredients (a.i.s) is relevant for many areas of life-science. However, despite its importance in pharmacy, toxicology, epidemiology, environmental science, agrochemistry and many other fields there is no consensus on the correct definition of synergy.

This consensus would be very helpful as it is necessary to define a reference behaviour, namely the response of an ensemble of compounds acting independently, in order to classify the observed effects as synergistic or antagonistic.

Hence, interaction-^1^ or combination-indices^2^, used to quantify the effects, are often based on differing definitions of additivity^3,4^.

The history of attempts to explain synergism was summarized in 1989 by the seminal work of Berenbaum^5^, followed by a series of related publications^3,4,6–13^, to quote only a few reviews.

Almost all approaches that describe synergy can be traced back to two competing assumptions on the action of drugs in mixtures, ADM and MSM^14^. ADM was introduced by Loewe^15^ and is based on the idea that an arbitrary dose of drug A in a mixture can be replaced by an iso-effective dose of another drug B in order to achieve the same effect. This is true if the doses are additive, with both drugs having parallel dose-response curves. As a consequence the effect of the mixture can not exceed that of its most potent ingredient. While the terms ‘effect summation’, ‘dose addition’, and ‘concentration addition’ are used synonymously for ADM, a better characterization of the model would be ’mutually exclusive action’^2,5^. In order to handle mixtures of partially overlapping agonists, the original ADM approach^1,15–17^ was extended and termed generalized concentration addition (GCA) model^8^.

Numerous response surface models^18–21^ are based on the concept of Loewe additivity. They avoid to describe synergism or antagonism of a mixture simply by a single number. It may happen that one dose-combination acts synergistic and another combination of the same drugs acts antagonistic. For that reason it is appropriate to look at response surfaces instead of considering distinct points on these surfaces or at isoboles, which are merely iso-effect lines on the surface.

A completely different ansatz is followed by the second approach, MSM. It is based on the definition of independence in probability theory and was introduced to agrochemical research by Abbott^22^, Limpel^23^ and Colby^24,25^ to classify mixture effects^26^. In toxicology this approach is known as Bliss independence^27,28^. The components of a mixture are assumed to act, mutually non-exclusively, by different modes of action. Hence, according to MSM the maximum possible effect is not limited by that of its most potent ingredient. Recently Bliss’ definition was extended to detect statistically significant synergy under various designs^29^. Astonishingly, by simply substituting Hill’s dose-response formulas into Colby’s formula under the assumption that all maximum effects are 100%, a new model name, ZIP (zero interaction metric) was created^30^!

Chou and Talalay’s median-effect models^2^ are derived from the law of mass-action. They belong to the most often used models in the literature^31,32^, although their mutually non-exclusive model is an *ad hoc* extension of the mutually exclusive one and has been criticized by several authors^5,7,18^ because of its questionable validity. A new mechanistic approach for binary mixtures, MuSyC^33,34^, is also based on the law of mass-action. It claims to unify different synergy metrics for binary mixtures and derives a Hill-like equation for a four-state state-transition model. In addition to the pure-compound parameters, two new parameters for each drug and one for their combination are introduced.

All of the approaches currently discussed are explanatory and not predictive, meaning that they are mainly used to fit experimental data. Response surface models are predictive only in the limited sense that they are able to find the optimum dose-ratio on a fitted surface. All models have inherent limitations: while the IA ansatz is not sham-compliant by design, Loewe additivity requires sham-compliance to be consistent. On the other hand, IA permits joint effects larger than the individual ones while GCA limits the maximum effect to be that of the most potent component. Mechanistic models are restricted to those mixtures which obey the proposed mechanism and are expected to become rather involved if they are extended to cover mixtures of more than two components.

Recently we presented an approach which describes dose-response surfaces of ’zero-interaction’ relying solely on the assumption that simultaneous administration of different drugs results in Hill-type shaped response surfaces^35^. While Hill’s equation^36,37^ can be obtained by solving a first-order ordinary differential equation (ODE), the logistic differential equation, its n-dimensional generalization results from solving a semilinear PDE. Its boundary conditions require that Hill’s equation results in the pure compound limit, and that the solution of the PDE is sham-compliant, meaning that an artificial partitioning of the one-dimensional problem into an n-dimensional one does not change the results.

In the present work we extend this ansatz to describe deviations from the reference surface by accounting for all n-tuples of drug interactions in n-component mixtures. It rests on the same principle, namely that the effect of both the pure compounds and their mixture can be described by Hill-type functions which are solutions of the underlying ODEs/PDEs with the appropriate boundary conditions. This defines - and limits - the application range of our approach. No further assumptions, neither on mechanisms of action nor on limitations of parameter combinations are necessary.

We introduce two types of parameters to the generalized Hill function, perturbations of the pure drug Hill parameters and interaction parameters that account for n-tuple interactions between all components of a mixture. The resulting ‘full-interaction’ response surface is a valid solution of the basic n-dimensional PDE, satisfying its boundary conditions. This is true irrespective of its actual functional form, as the number of parameters is not fixed within our framework. Starting from fitting the experimental data to the ‘full-interaction’ model with the maximum possible number of parameters, we gradually remove insignificant parameters until the optimum response surface model is obtained. Its quality is examined by the nonparametric Wilcoxon-Mann-Whitney (MW) test and the Akaike Information Criterion (AIC). The Shapiro-Wilk test is used to check for the normal distribution of the errors.

Comparison of ‘null-’ and ‘full-interaction’ surfaces permits the identification of synergistic or antagonistic effects for each point on the n-dimensional surfaces.

In addition we slightly extend our original ansatz to cover baseline responses different from zero, meaning that we replace the 3-parameter- by the 4-parameter Hill-equation as the basic dose-response curve. This is equivalent to moving from a logistic to the more general Riccati ODE. Consequently an n-component mixture is described by an n-dimensional Riccati-type PDE.

Verification and checking the boundary conditions, as well as performing the nonlinear fits und their statistical evaluation, is done by using Mathematica 12^38^.

In the next section we recapitulate our ’null-interaction’ model, slightly extended to cover baseline effects, and outline the ’full interaction’ formalism for binary and n-component mixtures. To facilitate the comparison with other approaches we translate our expressions of the two- and three-dimensional response surfaces into the notations used in the literature, followed by a short discussion of those response surface models that are used in the literature examples of the “Results” section. There we discuss three examples from different areas of life science. Our conclusions are given in the last section.

## Theoretical models

### Logistic functions and the Hill response surface

In^35^ we have discussed the close relation between Hill’s equation and the solution of the logistic ODE. Based thereon we proposed a null-interaction response surface model as a solution of the corresponding n-dimensional logistic PDE. While in^35^ we tacitly assumed that the baseline response was zero, this restriction will be removed in the present work. This means that we refer to the 4-parameter- instead of the 3-parameter Hill equation and solve Riccati-type ODEs and PDEs^39^ instead of logistic ones.

Denoting position and slope at the inflection point by $x_{50}$ and $\alpha$, the logistic function *a*(*x*) with minimum- and maximum effects $a_{min}$ and $a_{max}$ is

1 $$\begin{aligned} a(x) = a_{min} + \frac{a_{max}-a_{min}}{1+ e^{-\alpha \Delta x}} \end{aligned}$$

with $\Delta x = x - x_{50}$. Hill’s equation

2 $$\begin{aligned} E = E_0+\frac{E_{max}-E_0}{1+\left( \frac{EC_{50}}{C}\right) ^\alpha } \end{aligned}$$

and *a*(*x*) are intimatey connected. Actually, *x* is the natural logarithm of a dose with $-\infty \le x \le \infty$, whereas the dose itself (i.e., $e^x$ ) is $\ge 0$. Hence, we can identify the effects $E_0$, $E_{max}$ and the shape parameter $\alpha$ with $a_{min}$, $a_{max}$ and $\alpha$, and the doses *C* and $EC_{50}$ with $e^x$ and $e^{x_{50}}$ of Eq. (1), meaning that Hill’s 3- or 4-parameter equation is the solution of a logistic or Riccati ODE.

The Riccati-type PDE for the response surface of a binary mixture, characterized by slope $\gamma (x,y)$, minimum- and maximum-effect functions $u_{min}(x,y)$ and $u_{max}(x,y)$, is given by

3 $$\begin{aligned} u_{x} + u_{y} = \gamma \times \frac{\left( u-u_{min}\right) \left( u_{max} - u\right) }{u_{max}-u_{min}} \end{aligned}$$

where $u_x=\partial u(x,y)/\partial x$ and $u_y=\partial u(x,y)/\partial y$ denote the partial derivatives of *u*. Completely analogous to^35^, a solution of Eq. (3) is the ’null-interaction’ surface

4 $$\begin{aligned} u_{Hill}(x,y)= & {} u_{min} + \frac{u_{max}-u_{min}}{1+ \left[ e^{\Delta {x}} + e^{\Delta {y}}\right] ^{-\gamma }} \end{aligned}$$

with the ansatz

5 $$\begin{aligned} \gamma (x,y)= & {} \frac{\alpha e^{\Delta x} + \beta e^{\Delta y}}{e^{\Delta x}+e^{\Delta y}} \end{aligned}$$

6 $$\begin{aligned} u_{max}(x,y)= & {} \frac{a_{max} e^{\Delta x} + b_{max} e^{\Delta y}}{e^{\Delta x}+e^{\Delta y}} \end{aligned}$$

7 $$\begin{aligned} u_{min}(x,y)= & {} \frac{a_{min} e^{\Delta x} + b_{min} e^{\Delta y}}{e^{\Delta x}+e^{\Delta y}} \end{aligned}$$

While formally $u_{min}(x,y)$ is permitted to be a function of the a.i.s, for physical reasons $u_{min}$ is constant, as the baseline response, i.e., the response in the absence of a drug, is independent of any a.i.. Similar to the GCA approach, in the “null-interaction” model the maximum effect achievable by a mixture is that of its most potent component.

### The ’full-interaction’ response surface

Although one may doubt whether a perturbed response surface should be required to be a solution of a PDE at all, we stay within the framework of solving the basic PDE and thereby remove some arbitrariness in the descriptions of co-operative effects. Our approach starts from the ’null-interaction’ surface $u_{Hill}(x,y)$ and systematically accounts for deviations from this reference.

We use the fact that $u_{Hill}$ as given by Eq. (4) with the definitions of $\gamma$, $u_{max}$ and $u_{min}$ is not the only possible solution of Eq. (3). Modifications of these functions can be shown to be also solutions, if they do not violate the co-domain $0 \le u(x,y) \le 1$ of the response function and if the ’null interaction’ function *u*(*x*, *y*) is obtained in the limit of vanishing perturbations. However, whether the perturbed surface has to satisfy all the boundary conditions imposed on the reference surface, needs to be discussed. While the surfaces must be asymptotically correct for vanishing and infinitely high doses, sham-compliance might not be essential for the perturbations.

Two types of parameters are introduced: perturbations of the pure compound Hill parameters allow for individual changes of slopes, maximum effects and inflection points. For binary and ternary mixtures additional terms describe drug-drug- and three-drug-interactions, respectively. An extension to n-component mixtures is straightforward.

The basic idea of this approach is to maintain the functional form of the ’zero interaction’ surface and to augment it by formally similar functions. In addition to the unperturbed expressions these contain nth roots of products of n doses when they are used to describe n-component mixtures. This guaranties that the ’full interaction’ surface is a valid solution of the PDE. In order to keep the theory simple we do not consider other possible types of interactions, namely cross-terms between and among the perturbation- and interaction-terms mentioned above.

### Perturbations and interactions in binary mixtures

Co-operative effects in a mixture will lead to changes of the surface parameters. These include both perturbations $\delta a_{max}$, $\delta b_{max}$, $\delta x_{50}$, $\delta y_{50}$, $\delta \alpha$ and $\delta \beta$ of the Hill-parameters, and drug-drug interaction terms of the form $p \times e^{\frac{\Delta x +\Delta y}{2}}$, $(p= \delta {xy},\; \delta {\alpha \beta }, \;\delta ab =\delta a_{max}b_{max})$. As mentioned above we explicitly exclude terms like $p=\delta x\alpha ,\; \delta y b,\; \delta \alpha x_{50}$, etc. The baseline effect $u_{min}$ is assumed to be not affected by co-operative effects. Then the full-interaction response surface, solving Eq. (3), is

8 $$\begin{aligned} u_{Ricc}(x,y) = u_{min}+\frac{u_{max}+\delta u_{max}}{1+ \left[ e^{\Delta {X}}+\delta {xy} \times e^{\frac{\Delta {X} +\Delta {Y}}{2}} + e^{\Delta {Y}}\right] ^{-(\gamma +\delta \gamma )}} \end{aligned}$$

where we replaced $\gamma$ by $\gamma +\delta \gamma$, $u_{max}$ by $u_{max}+\delta u_{max}$ and introduced the definitions

9 $$\begin{aligned} \Delta {X}= & {} \Delta {x} - \delta x_{50} \text { and }\ \ \; \Delta {Y} = \Delta {y} - \delta y_{50} \end{aligned}$$

10 $$\begin{aligned} \delta u_{max}(x,y)= & {} \frac{\delta a\times e^{\Delta X} + \delta ab \times e^{\frac{\Delta X +\Delta Y}{2}}+ \delta b\times e^{\Delta Y}}{e^{\Delta X }+e^{\Delta Y }+e^{\frac{\Delta X +\Delta Y}{2}}} \end{aligned}$$

11 $$\begin{aligned} \delta \gamma (x,y)= & {} \frac{ \delta \alpha \times e^{\Delta X} + \delta {\alpha \beta }\times e^{\frac{\Delta X +\Delta Y}{2}}+ \delta \beta \times e^{\Delta X }}{e^{\Delta X }+e^{\Delta Y }+e^{\frac{\Delta X +\Delta Y}{2}}} \end{aligned}$$

while $u_{min}$, $u_{max}$ and $\gamma$ are defined by Eqs. (5)–(7). As discussed before, the binary interaction terms are square roots of products of two doses, $\sqrt{e^{\Delta x} e^{\Delta y}}=e^{\frac{\Delta x +\Delta y}{2}}$, and appear both in the denominators and the numerators of Eqs. (8)–(11). This ansatz can be verified by substituting Eqs. (8)–(11) into Eq. (3). In order to avoid mixing of different types of parameters and thus to facilitate the interpretation of co-operative effects we add the interaction parameter $\delta {xy}$ only to the numerator of Eq. (8). While in the pure-compound limit all interaction terms vanish automatically, the perturbation parameters have to be explicitly set to zero. Otherwise we would get

12 $$\begin{aligned} u(x,-\infty ) = \frac{a_{max}+\delta a}{1+ e^{-(\alpha +\delta \alpha ) (\Delta x - \delta x_{50})}} \;\; \text {and} \;\;\; u(-\infty ,y)= \frac{b_{max}+\delta b }{1+ e^{-(\beta +\delta \beta ) (\Delta y - \delta y_{50})}} \end{aligned}$$

and for infinitely high doses

13 $$\begin{aligned} u(\infty ,y) = a_{max} + \delta a \; \ \ \ \ \text {and } \;\; \;\; u(x,\infty )= b_{max} + \delta b \end{aligned}$$

To exclude physically impossible responses, $\delta u_{max}$ can vary only under the restriction that $u_{max}+\delta u_{max}\le 1$.

### The ‘full-interaction’ response surface for mixtures of n components

An extension of our formalism to mixtures of n agents $A_i$ is straightforward. The corresponding Riccati-type PDE is

14 $$\begin{aligned} \sum \limits _{i=1}^n{u_{x_i}}= \gamma (\vec {x}) \frac{\left( u(\vec {x})-u_{min}(\vec {x})\right) \left( u_{max}(\vec {x})-u(\vec {x})\right) }{u_{max}(\vec {x})-u_{min}(\vec {x})} \end{aligned}$$

where $\vec {x} = (x_1,\ldots ,x_n)$, $u_{x_i}=\partial u(\vec {x})/\partial x_i$ and $\Delta {X_i} = \Delta {x_i} - \delta x_{i_{50}}$. Its solution describes an n-dimensional full-interaction response surface. In addition to the ’null-interaction’ terms it contains contributions from the perturbed Hill- and drug-drug interaction parameters up to n-compound interactions. Here again it is essential that n-tuple interactions are described by nth roots of products of n doses, multiplied by their respective parameters.

15 $$\begin{aligned} u(\vec {x})= & {} u_{min}(\vec {x}) + \frac{u_{max}(\vec {x})-u_{min}(\vec {x})}{1+ \left[ \sum \nolimits _{i=1}^n{ e^{\Delta {X_i}}} + \sum \nolimits _{i,j}^{j>i} \delta x_{ij} e^{\frac{\Delta {X_i}+\Delta {X_j}}{2}}+ \sum \nolimits _{i,j,k}^{k>j>i} \delta x_{ijk} e^{\frac{\Delta {X_i}+\Delta {X_j}+\Delta {X_k}}{3}}+ \cdots +\delta x_{ij..n} e^{\frac{\sum {\Delta {X_i}}}{n}} \right] ^{\gamma (\vec {x})}} \end{aligned}$$

with

16 $$\begin{aligned} \gamma (\vec {x})= & {} \frac{\sum \nolimits _{i=1}^n{\alpha _i e^{\Delta x_i}}}{\sum \nolimits _{i=1,n}{e^{\Delta x_i}}} +\frac{\sum \nolimits _{i=1}^n{\delta \alpha _i e^{\Delta X_i}}+\sum \nolimits _{i,j}^{j>i}{\delta \alpha _{ij} e{\frac{\Delta X_i+\Delta X_j}{2}}}+\cdots }{\sum \nolimits _{i=1}^n{e^{\Delta X_i}}+\sum \nolimits _{i,j}^{j>i}{e{\frac{\Delta X_i+\Delta X_j}{2}}}+\cdots + e^{\frac{\sum {\Delta {X_i}}}{n}}} \end{aligned}$$

17 $$\begin{aligned} u_{max}(\vec {x})= & {} \frac{\sum \nolimits _{i=1,n}{a_{{max}_{i}} e^{\Delta x_i}}}{\sum \nolimits _{i=1,n}{e^{\Delta x_i }}} +\frac{\sum \nolimits _{i=1}^n{\delta a_i e^{\Delta X_i}}+\sum \nolimits _{i,j}^{j>i}{\delta a_{ij} e{\frac{\Delta X_i+\Delta X_j}{2}}} +\cdots }{\sum \nolimits _{i=1}^n{e^{\Delta X_i}}+\sum \nolimits _{i,j}^{j>i}{e{\frac{\Delta X_i+\Delta X_j}{2}}}+\cdots + e^{\frac{\sum {\Delta {X_i}}}{n}}} \end{aligned}$$

Formally $u_{min}(\vec {x})$ can be defined analoguously to $u_{max}(\vec {x})$, however, for physical reasons $u_{min}(\vec {x})$ will be constant. If all $\delta _i$-terms vanish, the “null-interaction” surface results, and in addition

18 $$\begin{aligned} u(-\infty ,\ldots ,x_i,\ldots ,-\infty )=a_i(x_i) \end{aligned}$$

While the number of perturbation parameters increases linearly with the number *n* of mixture components, the number of possible interaction terms grows as $$\sum \nolimits _{k=2}^n \left( {\begin{array}{c}n\\ k\end{array}}\right)$$, the reason being that building *k*-tuples of interaction terms, i.e., binary $(k=2)$, ternary $(k=3)$, quarternary $(k=4)$ etc., out of *n* compounds scales as $$\left( {\begin{array}{c}n\\ k\end{array}}\right)$$. This means that the number of possible parameters increases from 9 for binary to 21 for ternary and to 45 for quarternary mixtures!

Other approaches would need even more parameters. For example, to describe a quarternary mixture by MuSyC^33^, $2^n(n+1)-3n-1=67$ parameters are required.

Here we see a great advantage of our ansatz over approaches with a fixed number of parameters. The functional form chosen for the ’full interaction’ surface guaranties that $u_{Ricc}$ remains a valid solution of the basic Riccati PDE, Eq. (14), irrespective of the number of model parameters used. Hence, the number of parameters selected for a final model in an actual investigation depends only on its fit-statistics.

## Comparison of response surface models

In our previous article on Hill-type response surfaces^35^ we made a detailed comparison of different approaches describing synergy. Therefore we recapitulate here mainly the response surface models based on Loewe-additivity and their predecessors. We start with a translation of our theory to the nomenclature used in the literature.

### Nomenclature of literature expressions

When we discussed the close connection of the logistic ODE with Hill’s equation, we found that the exponential functions $e^x$ and $e^{x_{50}}$ correspond to doses in Hill’s equation. Consequently expressions like $e^{\Delta x-\delta x_{50}}$ in $u_{Ricc}$ denote doses $d_{a}$ scaled by their median effect $d_{a_{50}}$ and modified by a perturbation $\delta _{a_{50}}$. With the definitions $m_{a}=d_{a}/(d_{a_{50}}\delta _{a_{50}})= e^{\Delta x-\delta x_{50}}$ and $m_{b} =d_{b}/(d_{b_{50}}\delta _{b_{50}})= e^{\Delta y-\delta y_{50}}$ the ‘full-interaction’ Hill response surface of a binary mixture is given by

19 $$\begin{aligned} u_{Ricc}= & {} u_{min} + \frac{u_{max}-u_{min}}{1+ \left( m_{a} +\delta m_{ab} \sqrt{m_a m_b}+m_{b}\right) ^{-\gamma }} \end{aligned}$$

with

20 $$\begin{aligned} u_{min}= & {} \frac{a_{min}m_{a}+b_{min}m_{b}}{m_{a}+m_{b}} \end{aligned}$$

21 $$\begin{aligned} u_{max}= & {} \frac{a_{max}m_{a}+b_{max}m_{b}}{m_{a}+m_{b}} + \frac{\delta a\; m_{a}+\delta b \; m_{b}+\delta ab \sqrt{m_a m_b}}{m_{a}+m_{b}+\sqrt{m_a m_b}} \end{aligned}$$

22 $$\begin{aligned} \gamma= & {} \frac{\alpha \; m_{a} + \beta \; m_{b}}{m_{a}+m_{b}} + \frac{\delta \alpha \; m_{a} +\delta \alpha \beta \sqrt{m_a m_b} + \delta \beta \; m_{b} }{m_{a} + m_{b}+\sqrt{m_a m_b}} \end{aligned}$$

with the modifications $\delta \alpha$, $\delta \beta$, $\delta a$, $\delta b$, $\delta m_a$, $\delta m_b$ of the individual Hill parameters and the interaction terms $\delta {ab}, \delta m_{ab}$ and $\delta {\alpha \beta }$.

For ternary mixtures of a, b and c we get (with $m_i= d_{i}/(d_{i_{50}}\delta _{i_{50}}$)

23 $$\begin{aligned}&u_{Ricc} =&u_{min} + \frac{u_{max}-u_{min}}{1+ \left[ \sum \nolimits _{i}^{a,b,c} {m_i}+\sum \nolimits _{i,j}^{a<b<c}{\delta m_{ij} \sqrt{m_i m_j}}+\delta m_{abc} \root 3 \of {m_a m_b m_c}\right] ^{-\gamma }} \end{aligned}$$

where in a self-explaining notation (setting $u_{min}=const.$)

24 $$\begin{aligned} u_{max}= & {} \frac{\sum \nolimits _{i}^{a,b,c} { i_{max}\; m_i}}{ \sum \nolimits _{i}^{a,b,c} {m_i}} +\frac{\sum \nolimits _{i}^{a,b,c} {\delta i\; m_i}+\sum \nolimits _{i,j}^{a<b<c}{\delta ij\; \sqrt{m_i m_j}}+ \delta abc\; \root 3 \of {m_a m_b m_c}}{ \sum \nolimits _{i}^{a,b,c} {m_i}+\sum \nolimits _{i,j}^{a<b<c}{ \sqrt{m_i m_j}}+ \root 3 \of {m_a m_b m_c}} \end{aligned}$$

25 $$\begin{aligned} \gamma= & {} \frac{\sum \nolimits _{i}^{a,b,c} { \alpha _i\; m_i}}{ \sum \nolimits _{i}^{a,b,c} {m_i}}+ \frac{\sum \nolimits _{i}^{a,b,c} {\delta \alpha _i\; m_i}+\sum \nolimits _{i,j}^{a<b<c}{\delta \alpha _{ij}\; \sqrt{m_i m_j}}+ \delta \alpha _{abc}\; \root 3 \of {m_a m_b m_c}}{ \sum \nolimits _{i}^{a,b,c} {m_i}+\sum \nolimits _{i,j}^{a<b<c}{ \sqrt{m_i m_j}}+ \root 3 \of {m_a m_b m_c}} \end{aligned}$$

$$u_{Ricc}$$ satisfies $$u\left( (n-m)\times d,(1-n)\times d,m\times d\right) =a(d)$$ with $$0 \le n+m \le 1$$.

### Empirical response surface models

The CA model of mutually exclusive action for two noninteracting isoactive drugs A and B, acting according to Hill dose-response functions with slopes $\gamma _a$ and $\gamma _b$, is given by

26 $$\begin{aligned} 1 = \frac{m_a}{\left( \frac{u}{a_{max}-u}\right) ^{1/\gamma _a}} + \frac{m_b}{\left( \frac{u}{b_{max}-u}\right) ^{1/\gamma _b}} \end{aligned}$$

Greco derived a model for two-agent combined action by adding an interaction term, parameterized by a factor $\alpha$. Assuming that the Hill-type dose-response curves of A and B differ only in the slope parameters, he gets^18^

27 $$\begin{aligned} 1 = \frac{m_a}{\left( \frac{u}{u_{max}-u}\right) ^{1/\gamma _a}} + \frac{m_b}{\left( \frac{u}{u_{max}-u}\right) ^{1/\gamma _b}}+ \alpha \frac{m_a m_b}{\left( \frac{u}{u_{max}-u}\right) ^{(1/2\gamma _a+1/2\gamma _b)}} \end{aligned}$$

Although analytical expressions for *u* can be obtained from Eqs. (26) and (27) only under the restrictions of either a fixed maximum effect $a_{max}=b_{max}$ and identical slope parameters $\gamma _a=\gamma _b$ or of different maximum effects and identical slopes of unity $\gamma _a=\gamma _b=1$, they are the starting points for several response surface models, e.g., the GCA expression^8^ from Eq. (26). It permits different maximum effects but is limited to $\gamma =1$.

28 $$\begin{aligned} u_{GCA}= & {} \frac{a_{max} m_{a}+b_{max} m_{b}}{1 + a_{max} m_{a}+b_{max} m_{b}} \end{aligned}$$

Hence, $u_{GCA}$ is a special cases of $u_{Hill}$. The same holds true for Chou and Talalay’s mutually exclusive model^2^. It was derived from the the median effect principle, assuming both a constant $u_{max}$ and $\gamma$

29 $$\begin{aligned} u_{Chou_{ex}}= & {} u_{max} \frac{\left( m_{a}+ m_{b} \right) ^\gamma }{1 + \left( m_{a} + m_{b} \right) ^\gamma } \end{aligned}$$

Their mutually non-exclusive model^2^ is an *ad hoc* extension of Eq. (29)

30 $$\begin{aligned} u_{Chou_{nex}}= & {} u_{max} \frac{\left( m_{a}+ m_{b} + m_a m_b\right) ^\gamma }{1 + \left( m_{a}+ m_{b} + m_a m_b\right) ^\gamma } \end{aligned}$$

Minto^19^ proposed a model that solved the problem of the different denominators in Eq. (26) by expanding $u_{max}$ and $\gamma$ in polynomials in a parameter $\Theta$. Fidler^20^ extended Minto’s approach by adding an interaction term. Their model is

31 $$\begin{aligned} \begin{aligned} u(\Theta _p)&= \frac{u_{max}(\Theta _p) \left[ m_{a}+m_{b} + \alpha \times f \times \sqrt{m_a m_b}\right] ^{\gamma (\theta _p)}}{1+\left[ m_{a}+m_{b} + \alpha \times f \times \sqrt{m_a m_b}\right] ^{\gamma (\theta _p)}} \\ \theta _p&= \frac{m_a}{m_a + m_b} \end{aligned} \end{aligned}$$

where $\alpha$ indicates the type of interaction. Minto’s model corresponds to $\alpha =0$, $\alpha >0$ means synergism and $\alpha < 0$ antagonism. $f(s,w,\Theta _p)$ resembles a generalized $\Gamma$-distribution, and $u_{max}(\Theta _p)$ and $\gamma (\Theta _p)$ are functions of the potency fraction $\Theta _p$ and $f(s,w,\Theta _p)$. $\Theta _p$ ranges from 0 (drug A only) to 1 (drug B only). The key difference between our full-interaction $u_{Ricc}$ model with individual perturbation- and interaction terms and Fidler’s ansatz is the function $f(s,w,\Theta _p)$, modifying $\gamma$ and $u_{max}$ and thus accouunting for deviations from null-interaction. By truncating their polynomial ansatz for $u_{max}$ and $\gamma$ after the linear terms in $\Theta _p$, we have

32 $$\begin{aligned} \gamma (\Theta _p)= & {} \alpha \Theta _p + \beta (1-\Theta _p) = \gamma _{Hill} \end{aligned}$$

33 $$\begin{aligned} u_{max}(\Theta _p)= & {} a_{max} \Theta _p + b_{max} (1-\Theta _p) = u_{max} (Hill) \end{aligned}$$

Thus by making $\gamma (\Theta _p)$ and $u_{max}(\Theta _p)$ symmetric with respect to $m_a$ and $m_b$, $u_{Minto}$ becomes identical to $u_{Hill}$. However, inclusion of higher powers of $\Theta _p$ leads to violations of the boundary conditions and the sham compliance requirement. The flexible interaction model for three agents is somewhat lengthy and is described in appendix 1 of^20^.

## Results and discussion

In order to validate a theoretical synergy model it is necessary to test it using data sets with a sufficiently large number of experimental data. The paucity of data may lead to difficulties when trying to fit a multi-parameter function to few experimental mixture data. The fit problem becomes even worse for mixtures of more than two compounds and becomes really challenging for $n>3$. Fortunately, as shown in the examples below, very often the number of fit parameters can be reduced considerably without affecting the quality of the model.

Another problem concerns the error bars of the experiments. Often they are large or unknown. Let us assume that the experimental uncertainty of a dose-response curve from *in vivo* data is approximately 5$\%$, then the uncertainty of measuring a synergistic effect amounts to 15$\%$, as 3 sets of measurements are required, those of the pure components and of the mixture. Hence, when claiming synergy one needs effects greater than $\approx 15 \%$ to be on the safe side.

We choose three examples of increasing complexity that provide adequate numbers of mixture data, starting with the Yonetani and Theorell^40^ data already used by Chou^31^, followed by two examples of Short^41^ and Schlezinger^42^ from our previous publication^35^. The datasets analysed during the current study are available from Table 9 of^31^, listed in Table 2^41^ below, and from the Supplemental Material of^43^(doi:10.1289/ehp.0901312), respectively.

**Table 1:** Fit characteristics and parameters of anesthetics and their mixtures.

Drug	Minto^19^, Fidler^20^			Hill$^{c}$		Riccati$^{d}$			Interaction terms$^{e}$			peak$^{f}$ synergy
	$D_{50}$ $^{a}$	$\alpha$ $^{b}$	AIC$^{g}$	RMSE$^{h}$	MW$^{i}$	RMSE	AIC	MW	$\delta ab$	$\delta \alpha \beta$	$\delta m_{ab}$	
Midazolam	0.144	4.8		0.054		0.053						
Propofol	1.078	11.1		0.035		0.035						
Alfentanil	0.093	5.7		0.069		0.069						
Midazolam + Propofol			− 58.97	0.387	0.13	0.043	− 60.54	0.65	0.14	− 12.89	1.11	65
Midazolam + Alfentanil			− 36.33	0.477	0.21	0.051	− 37.64	0.97	− 0.08	1.28	1.68	66
Propofol + Alfentanil			− 35.09	0.228	0.37	0.053	− 42.68	0.63	0.35	− 12.34	0.38	37
Ternary mixture$^{j,k,l}$			− 106.75	0.461	0.09	0.046	− 121.06	0.79	0.00	2.46	− 1.16	68
$^{j,k}$									0.13	− 12.84	1.11	
$^{j,l}$									− 0.09	1.56	1.69	
$^{k,l}$									0.23	− 11.78	0.40	

$^{{\text {a}}}$ $D_{50}(mg/kg)$, fraction of dose causing hypnosis in 50% of the population.

$^{{\text {b}}}$Slope of the dose-response curve.

$^{{\text {c}}}$Null-interaction model, Eq. (4).

$^{{\text {d}}}$Full interaction model, Eq. (35).

$^{{\text {e}}}$Between maximum effects $\delta ab$, slopes $\delta \alpha \beta$ and inflection points $\delta m_{ab}$ ($\delta abc$, $\delta \alpha \beta \gamma$, $\delta m_{abc}$ for ternary mixture)

$^{{\text {f}}}$Calculated effect-difference between full- and null-interaction models, [%].

$^{{\text {g}}}$Goodness of fit according to the Akaike Information Criterion.

$^{{\text {h}}}$RMSE = root-mean-square error with respect to exptl. data.

$^{{\text {i}}}$p-values from the Wilcoxon-Mann-Whitney test.

$^{{\text {j,k,l}}}$Propofol, Midazolam, Alfentanil.

They serve to check whether our approach is flexible enough to cover a large variety of experimental responses and whether - and to what extent - the number of model parameters can be reduced without loosing accuracy in reproducing the response surface. The quality criteria used are the nonparametric MW, the AIC, the root-mean square error (RMSE) of the fit and the Shapiro-Wilk goodness-of-fit test for the normal distribution of the errors.

In the following sections we denote the null-interaction reference surface by $u_{Hill}$, the full-interaction surfaces by $u_{Ricc}$, and the difference $u_{Ricc}-u_{Hill}$ is assumed to be the synergy surface.

The general procedure is as follows: In a first step the null-interaction reference surface $u_{Hill}$ is generated, based on the Hill parameters of the pure compounds. Subsequently the experimental data are fitted to the full-interaction response surface $u_{Ricc}$ under the constraints that the individual maximum effects $a_{max}+\delta a$ etc. do not exceed $100\%$.

This is an iterative process. The nonlinear fit normally starts with the full model, subsequently removing those parameters whose coefficients are negligible and only marginally affecting the fit-statistics. The final form of $u_{Ricc}$ with a possibly much smaller number of parameters than the full-interaction model is chosen based on the respective fit-statistics.

Here we use the AIC ($AIC = 2 n - 2\ln L$) which provides a balanced score between goodness of fit (maximum value *L* of the likelihood function) and simplicity (number of parameters *n*) of the models under consideration. The nonparametric MW test is used to check that both experimental and modeled data describe the same distribution.

The resulting synergy surface, $u_{Ricc}-u_{Hill}$, is visualized by contour plots covering the dose-ranges of the mixture partners. Maxima and minima of this surface correspond to dose-combinations leading to peak synergism or antagonism.

The origin of the deviations from the null-interaction surface, i.e., the answer to the question which type of interaction causes the observed effect, can be found by looking at the magnitude of the individual perturbation/interaction terms.

### Alcohol dehydrogenase inhibitors o-phenanthroline and adenosine diphosphate ribose

We use experimental liver AdH inhibitor data of Yonetani and Thorell^40^ as an introductory example. They used a graphical method to examine the interaction of two competitive inhibitors at an enzyme and to obtain their kinetic constants. Fitting the 25 data points to a zero-interaction $u_{Hill}$ results in an adjusted $r^2$ of 0.9929 and an RMSE of 5.67$\%$. Inclusion of one interaction term, $\delta ab$, simplifies Eq. (19) considerably (Eq. 34) and leads to $r^2=0.9994$ and to a reduction of the RMSE to 1.82%, and if we additionally permit $\delta m_{ab}$ and $\delta \alpha \beta$ to contribute, we get an $r^2=0.9999$ and an error of 0.82$\%$, while the full model reveals an error of 0.69$\%$.

34 $$\begin{aligned} u_{Ricc} = \frac{u_{max}+\delta u_{max}}{1+ \left( m_{a} +m_{b}\right) ^{-\gamma }} { ; }\; \delta u_{max} = \frac{\delta ab \sqrt{m_a m_b}}{m_{a}+m_{b}+\sqrt{m_a m_b}} { ; } \delta \gamma =0 \end{aligned}$$

**Figure 1:**
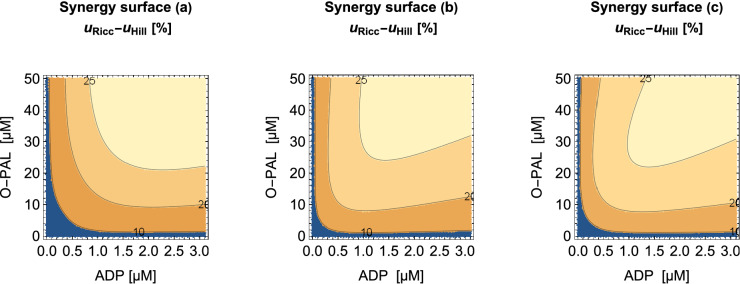
Contour plots of ADH inhibitor synergy surfaces $u_{Ricc} - u_{Hill}$ (in %) using different parameter sets for $u_{Ricc}$. The $u_{Ricc}$ surfaces contain (**a**) $\delta ab$-only, (**b**) all interaction parameters $\delta ab$, $\delta m_{ab}$ and $\delta \alpha \beta$, and (**c**) the full-parameter set.

On contour plots of synergy surfaces based on the three models (Fig. 1) we see that synergistic effects increase with increasing doses and exceed 25$\%$ . The differences between the one- (Eq. (34)), three- and the 9-parameter full Riccati surfaces (Eq. (19)) are rather small, indicating that one interaction parameter only, $\delta ab$, is sufficient to account for the main synergistic effect. According to its AIC the best model is the 3-interaction-parameter model b) of Fig. 1 with an AIC of − 228.5, as compared to − 177.1 for a) and − 223.8 for c), corroborated by the MW test providing p-values of 0.96, 0.99, and 0.96 for the one-, three-, and 9-parameter models. This demonstrates the flexibility of the ansatz, showing that the number of parameters for an optimum fit may require much less parameters than the maximum possible one.

### Mixtures of anesthetics

Anesthetic mixtures^41^ have been analyzed in great detail by^20^ and^19^ to test their flexible interaction models. For the present purpose the data for midazolam, propofol, and alfentanil, ranging from 0 (no hypnosis) to 1 (full hypnosis), were fitted to Hill dose-response curves and used to predict binary- and ternary mixture effects. The results are summarized in Table 1.

**Table 2:** Anesthetic mixtures: Exptl. data vs. ’full’- and ’null’-interaction models.

Midazolam [mg/kg]	Propofol [mg/kg]	Alfentanil [mg/kg]	exptl.$^{a}$ Effect	Fit results, based on parameters from:		
				Riccati$^{b}$ 3-dim	Riccati$^{b}$ 2-dim.	Hill$^{c}$ No interaction
0.1	0	0	0.2	0.1		0.1
0.125	0	0	0.3	0.3		0.3
0.15	0	0	0.5	0.5		0.5
0.175	0	0	0.8	0.7		0.7
0.2	0	0	0.8	0.8		0.8
0	0.7	0	0.1	0		0.
0	1	0	0.3	0.3		0.3
0	1.3	0	0.9	0.9		0.9
0	1.6	0	1	1		1
0	1.9	0	1	1		1
0	2.2	0	1	1		1
0	2.5	0	1	1		1
0	0	0.05	0	0.1		0
0	0	0.075	0.3	0.2		0.2
0	0	0.1	0.5	0.6		0.6
0	0	0.125	0.9	0.8		0.8
0	0	0.15	1	1		0.9
0.03	0.21	0	0.2	0.2	0.2	0.0
0.04	0.29	0	0.4	0.4	0.4	0.0
0.05	0.36	0	0.5	0.6	0.6	0.0
0.065	0.46	0	0.8	0.8	0.3	0.3
0.085	0.6	0	1	0.9	0.8	0.7
0.1	0.71	0	1	1	0.9	0.9
0.13	0.92	0	1	1	1	1.0
0.17	1.2	0	1	1	1	1.0
0	0.25	0.025	0.1	0.1	0.0	0.0
0	0.31	0.031	0.3	0.2	0.2	0.0
0	0.4	0.04	0.4	0.5	0.6	0.1
0	0.5	0.049	0.8	0.7	0.8	0.5
0	0.63	0.061	0.9	0.9	0.9	0.9
0.035	0	0.025	0.4	0.4	0.0	0.0
0.044	0	0.031	0.7	0.7	0.2	0.1
0.056	0	0.04	0.9	0.9	0.6	0.3
0.07	0	0.049	0.9	0.9	0.8	0.5
0.085	0	0.061	1	1	0.9	0.8
0.023	0.17	0.016	0.3	0.3		0.
0.03	0.21	0.021	0.6	0.6		0.
0.037	0.26	0.026	0.8	0.8		0.1
0.047	0.33	0.032	0.9	0.9		0.5
0.059	0.42	0.041	1	1		0.8

$^{\text {a}}$Data from^41^, 0 = no hypnosis, 1 = full hypnosis.

$^{\text {b}}$Full-interaction model $u_{Ricc}$, Eqs. (8) and (23), interaction parameters from Table 1.

$^c$ Hill’s equation for pure compounds, null-interaction model $u_{Hill}$ , Eq. (4), else.

It turns out that in this example the perturbation parameters are unimportant for the binary mixtures whereas all interaction parameters are needed. Hence, the number of parameters is reduced from 9 to 3 and Eq. (19) becomes Eq. (35)

35 $$\begin{aligned} u_{Ricc} = \frac{u_{max}+\delta u_{max}}{1+ \left( m_{a} +\delta m_{ab} \sqrt{m_a m_b}+m_{b}\right) ^{-(\gamma + \delta \gamma )}} \end{aligned}$$

with

36 $$\begin{aligned} \delta u_{max}= & {} \frac{\delta ab \sqrt{m_a m_b}}{m_{a}+m_{b}+\sqrt{m_a m_b}} \;\;\text { and }\;\; \delta \gamma = \frac{\delta \alpha \beta \sqrt{m_a m_b} }{m_{a} + m_{b}+\sqrt{m_a m_b}} \end{aligned}$$

While the RMSEs of fits are approximately 5% for the pure compounds, those for mixture predictions using $u_{Hill}$ are of the order of 40%. However, using $u_{Ricc}$ leads to a reduction of the RMSEs by factors of $\approx 10$, and a comparison of the p-values from the MW tests for the null- and the full-interaction models reveals that the Hill surfaces do not adequately describe the binary and ternary response surfaces.

**Figure 2:**
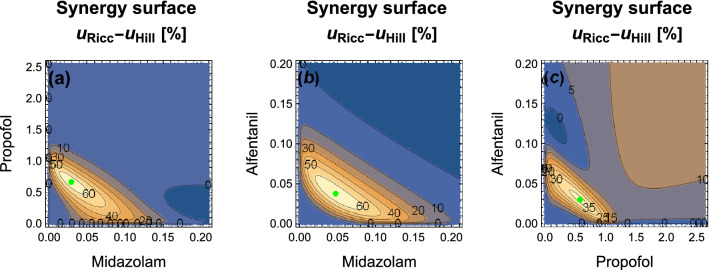
Synergy surfaces for binary mixtures of anesthetics, based on $u_{Ricc}$ of Eq. (35), with parameters $\delta ab$, $\delta \alpha \beta$ and $\delta m_{ab}$ from Table 1. Doses in [mg/kg]. Green dots denote dose combinations leading to peak synergistic effects.

Comparing the AICs of the best Fidler/Kern response surface models (Table 1 of^20^) with our results (Table 1), we find only marginal differences for the two-drug models midazolam/propofol and midazolam/alfentanil, but for propofol/alfentanil and for the three-drug case AIC differences of $-7.59 (=-42.68+35.09)$ and $-14.31 (=-121.06+106.75)$ show strong to very strong evidence in favour of the Riccati models. Especially large are changes due to slope-interaction $\delta \alpha \beta$. The resulting synergy surfaces (Fig. 2) show regions with synergistic effects exceeding 60%.

**Table 3:** AhR agonist parameters from GCA- and Hill-models.

Ligand	GCA, $\gamma = 1$^42^		Hill, $\gamma$ variable		
	$E_{max}(\%)$	$EC_{50}(M)$	$E_{max}(\%)$	$EC_{50}(M)$	$\gamma$
TCDF$^{b}$	100	$2.9\times 10^{-11}$	100	$3.2\times 10^{-11}$	0.88
PCB126$^{c}$	99	$4.1\times 10^{-10}$	100	$4.5\times 10^{-10}$	0.82
TCDD$^{d}$	100	$7.6\times 10^{-12}$	100	$6.3\times 10^{-12}$	1.29
PCB105	61	$1.4\times 10^{-6}$	56	$9.2\times 10^{-7}$	1.45
TCDD	100	$9.9\times 10^{-12}$	100	$8.5\times 10^{-12}$	1.09
Galangin	30	$4.1\times 10^{-6}$	35	$4.7\times 10^{-6}$	0.79
TCDD	100	$9.1\times 10^{-12}$	100	$6.5\times 10^{-12}$	1.22
DIM$^{e}$	8	$6.6\times 10^{-6}$	10	$8.5\times 10^{-6}$	1.62

Parameters are slopes $\gamma$, maximum effects $E_{max}$ and $EC_{50}$ values of the agents.

$$^{\text {b}}$$2,3,7,8-tetrachlorodibenzofuran;

$$^{\text {c}}$$2,3,3’,4,4’-petachlorobiphenyl;

$$^{\text {d}}$$2,3,7,8-tetrachlorodibenz-p-dioxin;

$$^{\text {e}}$$3,3’-diindolylmethane.

For the ternary mixture all perturbation terms disappear in the final model, while all two- and two of the three-compound interactions ($\delta \alpha \beta \mu$ and $\delta m_{abc}$) are important. This means that $\delta i=\delta \alpha _i=0$ and $\delta m_i =1\; \forall i \in a,b,c$ in Eq. (25), and the number of parameters is reduced from 21 to 11. It is interesting to note that coefficients of the binary interaction terms are almost transferable to their analogs in the ternary mixture. Similar findings were reported by^20^. In Table 2 we compare experimental data with results from fits using $u_{Hill}$ (parameter sets from the pure compounds) and from two full-interaction models $u_{Ricc}$, based on Eqs. (8) and (23), respectively. For all ’full-interaction’ models the null hypothesis that the errors are distributed according to the Normal Distribution is not rejected at the 5% level based on the Shapiro-Wilk test.

**Table 4:** AhR agonist parameters for ’null’- and ’full’-interaction surfaces.

Mixture	Set	Perturbation terms$^{a}$						Interaction terms$^{b}$			RMSE$^{c}$	AIC$^{d}$
		$\delta a$	$\delta b$	$\delta m_a$	$\delta m_b$	$\delta \alpha$	$\delta \beta$	$\delta {ab}$	$\delta {\alpha \beta }$	$\delta m_{ab}$		
TCDF$^{e}$ + PCB126^f^	0	–	–	–	–	–	–	–	–	–	4.5	
	1”	–	–	–	–	− 0.15	0.02	–	–	–	3.6	− 157.5
	1’	–	–	1.05	0.91	− 0.13	− 0.01	–	–	–	3.5	− 155.8
	1	− 0.05	0.00	0.92	0.91	− 0.04	− 0.01	–	–	–	3.4	− 153.2
	2	–	–	–	–	–	–	0.01	− 0.37	0.18	3.8	− 151.1
	3	–	–	–	–	–0.13	0.05	0.04	− 0.40	0.17	3.6	− 152.8
	4	–	–	1.08	0.94	− 0.11	0.03	0.02	− 0.32	0.157	3.5	− 149.9
	5	− 0.05	0.00	0.93	0.93	− 0.01	0.02	0.06	− 0.36	− 0.01	3.4	− 146.8
TCDD$^{g}$ + PCB105^h^	0	–	–	–	–	–	–	–	–	–	8.0	
	1”	–	–	–	–	− 0.28	− 0.66	–	–	–	6.6	− 112.3
	1’	–	–	1.29	0.91	− 0.13	− 0.62	–	–	–	6.1	− 112.8
	1	0.0	− 0.10	1.22	0.61	− 0.10	0.09	–	–	–	5.0	− 126.6
	2	–	–	–	–	–	–	− 0.41	− 0.53	0.40	5.6	− 124.4
	3	–	–	–	–	0.01	− 0.33	− 0.38	− 0.26	0.25	5.5	− 121.2
	4	–	–	1.07	0.85	0.07	− 0.40	− 0.35	0.00	0.16	5.4	− 118.0
	5	− 0.05	0.00	0.93	0.93	− 0.01	0.02	0.06	− 0.36	− 0.01	4.5	− 130.8
TCDD + Galangin	0	–	–	–	–	–	–	–	–	–	7.1	
	1”	–	–	–	–	− 0.08	− 0.19	–	–	–	7.0	− 108.0
	1’	–	–	0.91	0.46	0.13	− 0.14	–	–	–	5.9	− 119.8
	1	0.0	− 0.06	1.13	0.57	0.03	2.52	–	–	–	5.5	− 121.9
	2	–	–	–	–	–	–	− 0.45	7.3	2.30	5.5	− 128.8
	3	–	–	–	–	0.15	− 0.34	0.62	− 2.85	4.85	5.1	− 130.3
	4	–	–	0.92	0.11	− 0.04	− 0.46	− 0.18	8.12	− 0.18	4.9	− 129.7
	5	0.0	− 0.02	0.50	− 0.43	− 0.04	− 0.35	− 0.10	8.73	− 0.41	4.9	− 125.7
TCDD + DIM^i^	0	–	–	–	–	–	–	–	–	–	14.2	
	1”	–	–	–	–	− 0.50	− 1.32	–	–	–	9.9	− 83.8
	1’	–	–	1.10	0.17	0.28	− 0.19	–	–	–	4.0	− 181.4
	1	0.0	− 0.01	1.08	0.17	0.24	0.01	–	–	–	4.0	− 177.3
	2	–	–	–	–	–	–	0.02	− 2.47	− 1.83	5.5	− 129.4
	3	–	–	–	–	0.20	− 0.21	− 0.05	− 2.74	− 1.88	5.3	− 146.2
	4	–	–	0.92	0.11	− 0.09	− 0.94	0.16	1.54	− 0.66	3.8	− 181.8
	5	0.0	− 0.05	1.00	0.10	0.28	0.78	0.31	− 0.85	− 0.58	3.7	− 178.5

$^{\text {a}}$Perturbation of maximum effects $\delta a$,$\;\delta b$, inflection points $\delta m_a$,$\;\delta m_b$ and slopes $\delta \alpha$,$\;\delta \beta$, $\delta m_{a}= e^{\delta x_{50}}=1$ in Eq. (19) corresponds to $\delta x_{50}=0$, the same holds true for $\delta m_b$.

$^{\text {b}}$Interaction between maximum effects $\delta {ab}$, slopes $\delta {\alpha \beta }$ and inflection points $\delta m_{ab}$.

$^{\text {c-d}}$See legends $^{g-h}$ of Table 1.

$^{\text {e-i}}$See legends $^{b-e}$ of Table 3.

Peak synergies of increasing magnitude, marked with green dots in Fig. 2, are observed for the binary mixtures of propofol/alfentanil (37% at doses of 0.575 and 0.0298 mg/kg), midazolam/propofol (65% at doses of 0.0292 and 0.667 mg/kg) and midazolam/alfentanil (66% at doses of 0.0474 and 0.03776 mg/kg). The synergistic effect of the ternary mixture (68%) is comparable to the best binary one. Again this is in line with the observation of refs.^19^ and^20^ , who find the same ranking of synergistic effects, although lower in magnitude (16%, 35%, and 44%^19^ as compared to 20%, 41% and 45%^20^), and note that no synergy beyond that expexted from the paired interactions was found.

**Figure 3:**
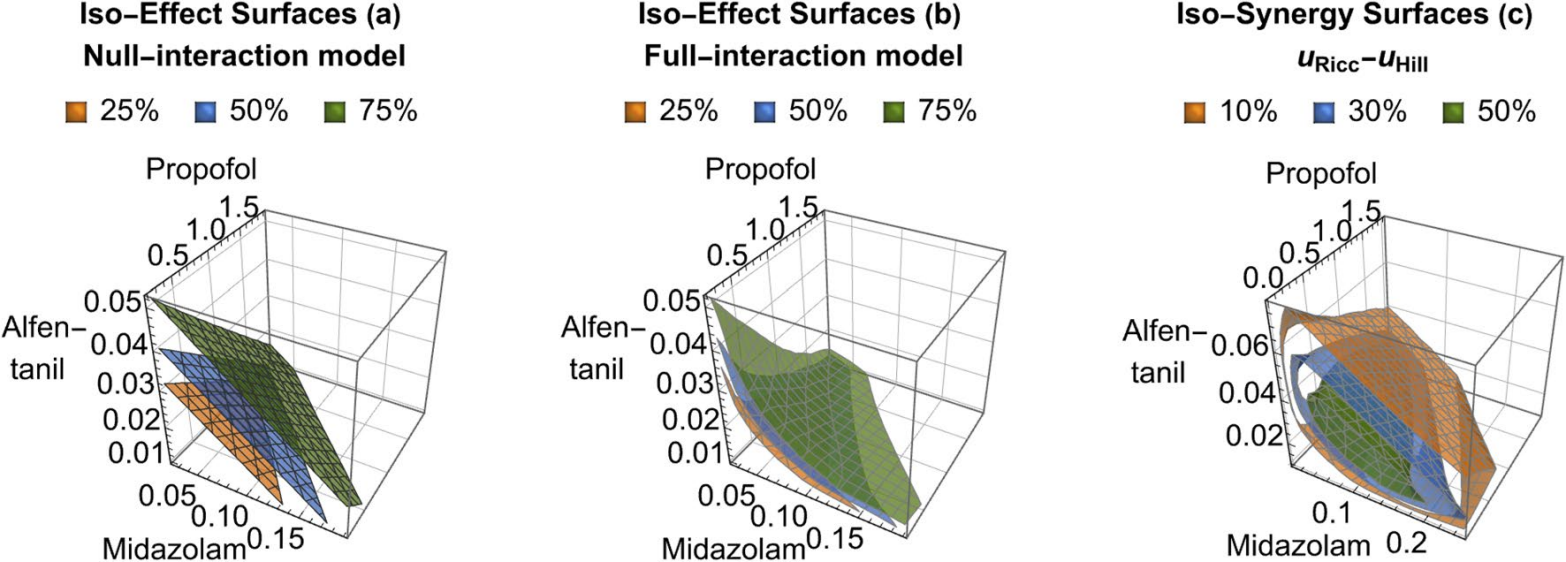
Iso-response surfaces for the ternary anesthetic mixture of propofol, midazolam and alfentanil at 25%, 50%, and 75% effect levels. Doses in [mg/kg]. Shown for the null-interaction (**a**) and the ’full-interaction’ (**b**) models. Their difference $u_{Ricc} - u_{Hill}$ (**c**) shows iso-synergy surfaces at 10%, 30% and 50% levels.

Figure 3 shows iso-effect surfaces for the ternary mixture at the 25$\%$, 50$\%$ and 75$\%$ effect levels for both models and their difference, the iso-synergy surfaces. As expected, the null-interaction model leads to planar iso-surfaces, similar to straight-line isoboles for binary mixtures, while they are curved in the full-interaction case. The maximum of the iso-synergy surfaces (68%) is observed at low doses of midazolam (0.032 mg/kg) and alfentanil(0.0116 mg/kg), combined with medium high doses of propofol(0.48 mg/kg).

**Figure 4:**
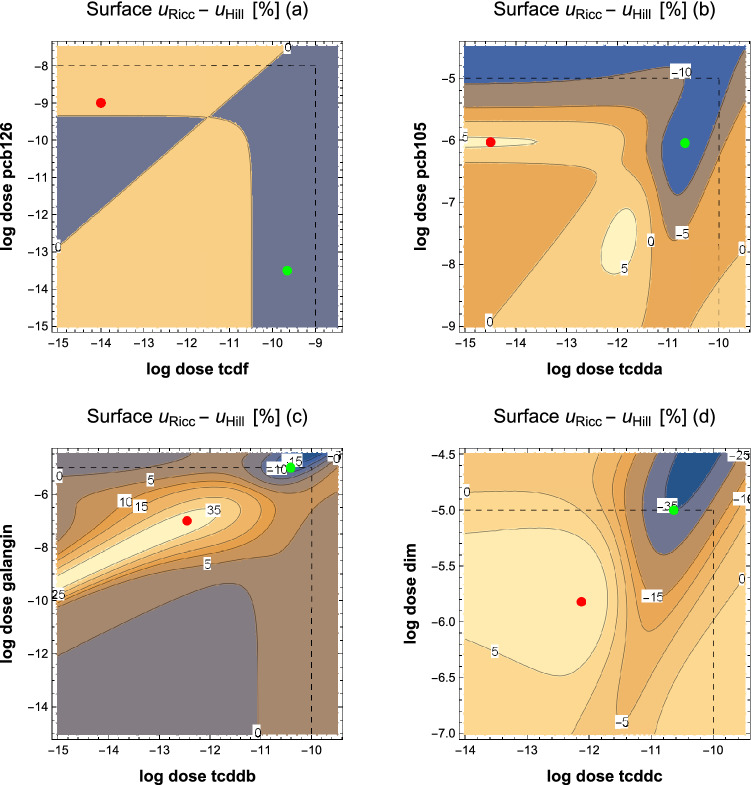
Synergy surfaces of binary AhR ligand mixtures (in %) , obtained as $u_{Ricc}-u_{Hill}$ using the respective optimum parameter set from Table 4, according to the AIC criterion. Dashed lines denote maximum doses used in the experiments. The observed responses range from null-interaction within exptl. error (**a**) TCDF+PCB126 (parameter set 1”) and (**b**) TCDD+PCB105 (parameter set 5) to simultaneous presence of synergism and antagonism (**c**) TCDD+Galangin (parameter set 3) and clear antagonism (**d**) TCDD+DIM (parameter set 4). Green an red dots refer to minima and maxima of the synergy surfaces.

**Table 5:** Minima and maxima of synergy surfaces: peak synergism/antagonism for AhR agonists.

Mixture	Peak dose combination		$E_{calc}$(%)		$\Delta E_{calc}$(%)$^{{\text {a,f}}}$				
	Dose a	Dose b	$E_0$	$E_5$	$\Delta E_{5}$	$\Delta E_{4}$	$\Delta E_{3}$	$\Delta E_{2}$	$\Delta E_{1}$
TCDF$^{b}$ + PCB126	$1.0\times 10^{-14}$	$1.0\times 10^{-09}$	66	68	2	2	7	2	5
	$1.0\times 10^{-09}$	$3.2\times 10^{-15}$	95	90	− 5	− 4	− 4	− 4	− 3
TCDD$^{d}$ + PCB105^c^	$1.0\times 10^{-14}$	$9.3\times 10^{-07}$	28	34	5	5	3	1	3
	$2.2\times 10^{-11}$	$9.0\times 10^{-07}$	79	65	− 15	− 14	− 14	− 13	− 13
TCDD + Galangin	$2.4\times 10^{-11}$	$3.8\times 10^{-07}$	71	85	14	13	40	7	22
	$4.4\times 10^{-12}$	$2.3\times 10^{-07}$	33	7	− 27	− 24	− 13	− 13	− 12
TCDD + DIM^e^	$8.1\times 10^{-13}$	$1.7\times 10^{-06}$	3	13	10	11	11	7	11
	$2.4\times 10^{-11}$	$1.0\times 10^{-05}$	73	38	− 35	− 35	− 36	− 35	− 30

$^{\text {a}}$Peak-difference between calculated full-($E_5$) and null-interaction ($E_0$) responses at the respective dose-combinations of $E_5$ (corresponding dose combinations not shown for the $E_1$ to $E_4$ surfaces). Subscripts denote the parameter sets from Table 4.

$^{\text {b}-\text{e}}$See legends of Table 3.

$^{\text {f}}$Values of $\approx 15 \ge \Delta E \ge -\approx 15$ are assumed to be within the exptl. errors.

### Dioxin-like chemicals

In^42^ Aryl hydrogen receptor (AhR) ligands were used to compare the toxic equivalence factor approach and the GCA ansatz to predict the effect of mixtures of full agonists (TCDD, TCDF) with agonists (PCB126), partial agonists (PCB105, Galangin) or antagonists (DIM). From their Supplemental Material^43^ Hill curves with variable $\gamma$ were derived and used to predict $u_{Hill}$. It turned out that the differences in $E_{max}$- and $EC_{50}$-values between $u_{GCA}$ (Eq. (28)) and $u_{Hill}$ are much smaller than the experimental error bars (c.f. Table 3). As $u_{GCA}$ is a special case of $u_{Hill}$, these differences result from fixing $\gamma$ in GCA $(\gamma =1)$ and $\gamma \ne 1$ in the Hill ansatz.

In Table 4 the variation of the quality of fits with the number and type of fit parameters is shown, starting from the null model (parameter set 0 in Table  4) and ending with the full model (Eq. (19), parameter set 5). As the GCA expression is a special case of $u_{Hill}$, and as the introduction of variable slopes for the pure compounds has only very minor effects (c.f. Table 3), the results referring to set 0 are equivalent to those from ref^42^.

We note that the choice of parameters affects the quality of the TCDF+PCB126 surface only marginally as it is already satisfactorily described by the null-model. Actually, the simplest interaction model with perturbations of the slope-parameters only turns of to be the best model with an AIC of − 157.5. Two mixtures, TCDD+PCB126 (AIC = − 126.6) and TCCD+DIM (AIC = − 177.3) are described fairly well by perturbation parameters only (parameter set 1), while inclusion of the interaction terms improves the model only slightly (AICs = − 130.8 and − 181.8 for sets 5 and 4, respectively). The situation is different for the TCDD+Galagin mixture, where interaction terms (AIC = − 128.8, set 2), augmented by slope perturbations (AIC = − 130.3, set 3) constitute the best models. It is interesting to note that the largest deviations from ’null-interaction’ are found for the agonist/antagonist mixture TCCD+DIM.

The superiority of the respective optimum Riccati models over the null-model is demonstrated by comparison of the p-values from nonparametric MW tests. We find 0.84 vs. 0.90 for the combination TCDF+PCB126, 0.35 vs. 0.94 for TCDDA+PCB105, 0.83 vs. 0.98 for TCDBB+galangin, and 0.39 vs. 0.89 for TCDDC+DIM, respectively. This comparison reveals also, that the null-model is able to describe the pair of full agonists, TCDF+PCB126, and the combination of a full agonist with a partial agonist of low efficacy, TCDBB+galangin. It is less appropriate for the description of the combinations of a full and a partial agonist, TCDD+PCB105, and a full agonist with a nearly complete competitive antagonist, TCDD+DIM.

Comparing our data with the MW statistics of Ref.^42^ we find p values for the rejection of their GCA model of 0.86, 0.63, 0.79, and 0.65. While they agree with our results for the pairs TCDF+PCB126 and TCDBB+galangin, they seem to be rather high for the remaining ones.

Neglecting changes of maximum effects ($\delta a=\delta b=0$) worsens the quality of fits only slightly. This might be due to the fact that their respective fit-coefficients are already rather small. The exception is the mixture TCDD+PCB105 with $\delta b=-0.1$. The statistics is hardly affected, too, when we also disregard changes in the inflection points. Here the exception is TCDD+DIM, whose RMSE becomes significantly worse. And finally, omission of all perturbation terms has a larger effect on the quality of fits than the omission of all interaction terms.

An analysis of the synergy surfaces (Fig. 4), based on the full interaction model (set 5), can locate their extrema and calculate the synergistic effect $u_{Ricc}-u_{Hill}$ at the respective dose-combinations $(d_a,d_b)$. These are marked by red and green dots in Fig. 4. Having in mind that in^42^ the authors observed an unexplained decline in reporter activity at the highest doses for some ligands, we restrict our regions of search to the experimentally observed dose ranges (dashed lines in Fig. 4). The differences between sets 1–5 of Table 4 in the prediction of maximum synergistic effects are shown in Table 5. In addition, the dose-combinations leading to the synergism-/antagonism-extrema according to parameter set 5 are given.

Three types of interaction can be distinguished: 1. no interaction within the experimental errors (TCDF+PCB126 and TCDD+PCB105), 2. presence of (borderline) synergism and pronounced antagonism (TCDD+Galangin), where antagonism is found at the highest doses of both mixture partners and synergism on a narrow band connecting the dose-combinations (− 12,− 7) and (− 15,− 9.5) on the logarithmic scale of Fig. 4c, and 3. clear antagonism (TCDD+DIM). As shown in Table 5 and in Fig. 4a–d, synergistic effects are within the experimental errors for all mixtures. Antagonism is found to be borderline for TCDD+PCB105 (− 14%), pronounced for TCDD+Galangin (− 27$\%$) and for TCDD+DIM (− 35$\%$). For TCDD+DIM the global minimum would be located at higher doses of DIM than applied in the experiments. The TCDD+Galangin mixture is special, as the resulting effects depend strongly on the parameter sets used to fit the experimental data. All other mixtures show roughly the same pattern of synergism and antagonism irrespective of using smaller ($\Delta E_{1,0}-\Delta E_{4,0}$) or larger ($\Delta E_{5,0}$) parameter sets.

## Conclusions

Our ansatz to describe synergistic effects rests on the assumption that the activity of both single drugs and drug-mixtures can be described by (n-dimensional) generalized Hill-type functions which are solutions of the underlying differential equations. While this simple concept is easily extended to mixtures of many components, it clearly restricts the range of applicability of our approach.

Based on the 4-parameter Hill equation for pure compounds analytical expressions for the null- and full-interaction response surfaces of n-component mixtures have been derived by solving the respective Riccati-type PDEs. Co-operative effects are handled by introducing perturbation- and interaction parameters in a systematic way. The resulting full-interaction models are also solutions of the PDE and fulfill the necessary boundary conditions. Synergistic or antagonistic effects are visualized and analyzed by (iso-)synergy surface plots.

Using examples from the literature we have illustrated how to identify those dose-combinations that cause maximum co-operative effects in a mixture. In one example from environmental science we found a binary mixture that exhibits both synergistic and antagonistic effects, depending on the dose-ratio of its ingredients.

Especially for n-component mixtures we see a main advantage of our approach over models with a fixed number of parameters, namely the flexibility of the n-dimensional Riccati response function in actual applications. Without loosing the requested property of being a valid solution of the PDE, the number of model parameters necessary to describe the experimental data can be minimized, solely based on the statistics of the nonlinear fits.

A major result of our investigations is the observation that parameter reduction is indeed possible without significant loss of accuracy. This facilitates the application of our ansatz to small sets of experimental data and simultaneously permits to focus on those parameters that are responsible for the observed co-operativity. Hence, an interpretation of deviations from the null-interaction surface is easily possible by looking at the magnitude of the perturbation- and/or interaction-coefficients of the model.

In the literature examples investigated the importance of the different types of model parameters varies. While modification of slopes and inflection points plays a major role and the introduction of interaction terms improves the statistics, changes of individual maximum effects seem to be of minor importance. However, these findings may change when the theory is applied to other examples.
